# Methyl (*E*)-2-cyano-3-(6-nitro-1,3-benzodioxol-5-yl)acrylate

**DOI:** 10.1107/S1600536812043164

**Published:** 2012-10-20

**Authors:** M. Bakthadoss, A. Devaraj, D. Lakshmanan, S. Murugavel

**Affiliations:** a Department of Organic Chemistry, University of Madras, Maraimalai Campus, Chennai 600 025, India; b Department of Chemistry, Pondicherry University, Puducherry 605 014, India; c Department of Physics, C. Abdul Hakeem College of Engineering & Technology, Melvisharam, Vellore 632 509, India; d Department of Physics, Thanthai Periyar Government Institute of Technology, Vellore 632 002, India

## Abstract

In the title compound, C_12_H_8_N_2_O_6_, the 1,3-benzodioxole ring system is essentially planar [maximum deviation = 0.036 (2) Å] and the nitro group is oriented at a dihedral angle of 15.4 (1)° with respect to its mean plane. In the crystal, moleucles are linked into *C*(8) [101] chains by C—H⋯O hydrogen bonds, and weak aromatic π–π stacking [centroid–centroid distance = 3.887 (1) Å] also occurs.

## Related literature

For a related structure and background references, see: Karthikeyan *et al.* (2011 ▶); Loghmani-Khouzani *et al.* (2009 ▶).
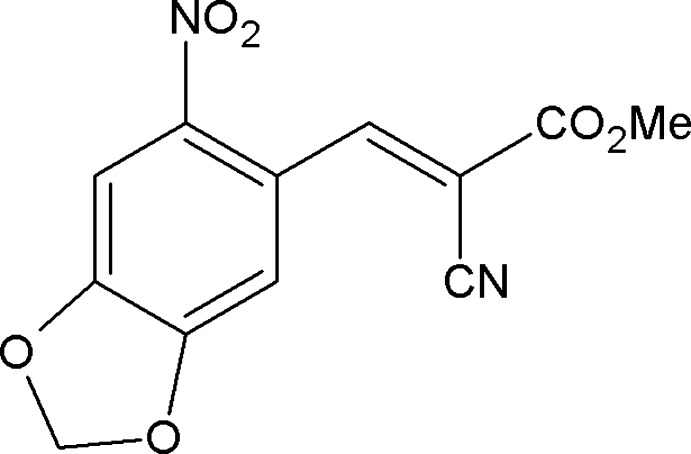


## Experimental

### 

#### Crystal data

C_12_H_8_N_2_O_6_
*M* *_r_* = 276.20
Monoclinic, 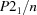

*a* = 10.8191 (9) Å
*b* = 7.3220 (6) Å
*c* = 15.4133 (13) Å
β = 91.691 (2)°
*V* = 1220.47 (18) Å^3^
*Z* = 4
Mo *K*α radiation
μ = 0.12 mm^−1^
*T* = 293 K
0.23 × 0.22 × 0.17 mm

#### Data collection

Bruker APEXII CCD diffractometer
Absorption correction: multi-scan (*SADABS*; Sheldrick, 1996 ▶) *T*_min_ = 0.972, *T*_max_ = 0.979
14190 measured reflections
3567 independent reflections
2286 reflections with *I* > 2σ(*I*)
*R*_int_ = 0.027

#### Refinement

*R*[*F* ^2^ > 2σ(*F* ^2^)] = 0.046
*wR*(*F* ^2^) = 0.129
*S* = 1.03
3567 reflections
182 parameters
H-atom parameters constrained
Δρ_max_ = 0.20 e Å^−3^
Δρ_min_ = −0.18 e Å^−3^

### 

Data collection: *APEX2* (Bruker, 2004 ▶); cell refinement: *APEX2* and *SAINT* (Bruker, 2004 ▶); data reduction: *SAINT* and *XPREP* (Bruker, 2004 ▶); program(s) used to solve structure: *SHELXS97* (Sheldrick, 2008 ▶); program(s) used to refine structure: *SHELXL97* (Sheldrick, 2008 ▶); molecular graphics: *ORTEP-3* (Farrugia (1997 ▶); software used to prepare material for publication: *SHELXL97* and *PLATON* (Spek, 2009 ▶).

## Supplementary Material

Crystal structure: contains datablock(s) global, I. DOI: 10.1107/S1600536812043164/hb6968sup1.cif

Structure factors: contains datablock(s) I. DOI: 10.1107/S1600536812043164/hb6968Isup2.hkl

Supplementary material file. DOI: 10.1107/S1600536812043164/hb6968Isup3.cml

Additional supplementary materials:  crystallographic information; 3D view; checkCIF report

## Figures and Tables

**Table 1:** Hydrogen-bond geometry (Å, °)

*D*—H⋯*A*	*D*—H	H⋯*A*	*D*⋯*A*	*D*—H⋯*A*
C3—H3*B*⋯O4^i^	0.97	2.51	3.247 (2)	132

Symmetry code: (i) 
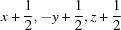
.

**Fig. 1.**
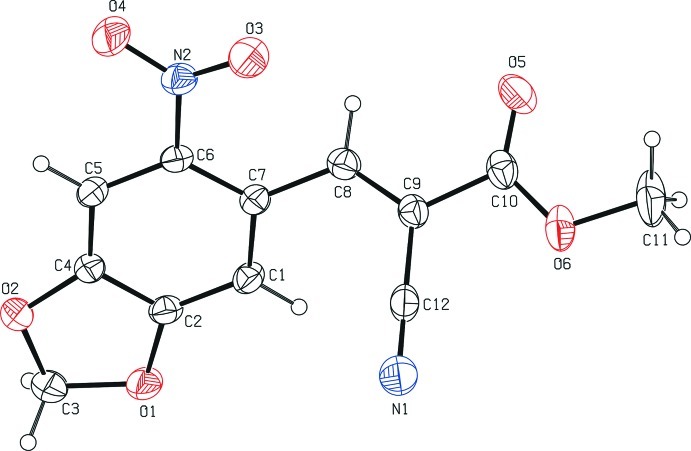
The molecular structure of the title compound with displacement ellipsoids drawn at the 30% probability level.

**Fig. 2.**
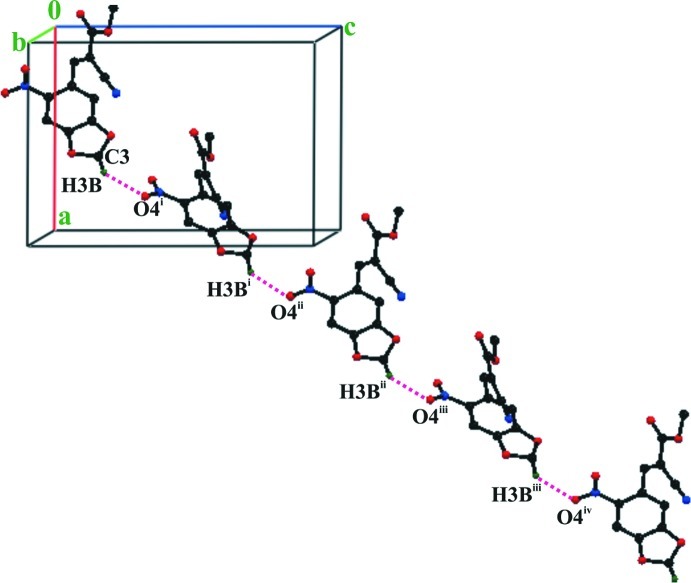
Part of the crystal structure of (I) showing C—H···O hydrogen bonds (dotted lines), with the formation of C(8) chains along [101]. [Symmetry codes: (i)1/2+x, 1/2-y, 1/2+z; (ii)1+x, y, 1+z; (iii)<3/2+x, 1/2-y, 3/2+z; (iv)2+x, y, 2+z].

**Fig. 3.**
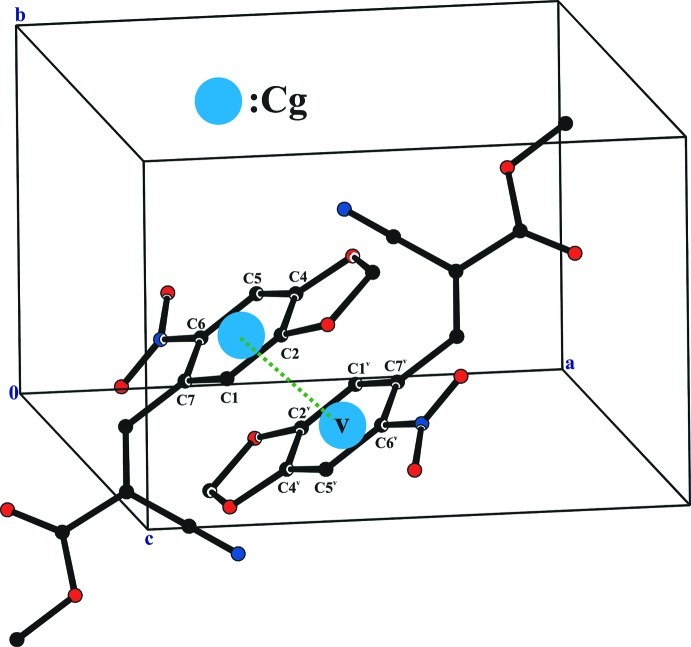
A view of π—π (green dotted lines) interactions in the crystal structure of the title compound. Cg denotes the centroid of the C1/C2/C4/C5/C6/C7 benzene ring [Symmetry code:(v)1-x, 1-y, -z].

## supplementary crystallographic information

### Comment

As part of our ongoing studies of benzodioxoles with possible biological activities (Karthikeyan *et al.,* 2011), the crystal structure of the title compound has been determined and the results are presented here.

Fig. 1. shows a displacement ellipsoid plot of (I), with the atom numbering scheme. The benzodioxole ring system is essentially planar [maximum deviation = 0.036 (2) Å for the C3 atom] and is oriented at a dihedral angle of 15.4 (1)° with respect to the nitro group. The sum of bond angles around N2 (360°) indicates that N2 is in *sp*^2^ hybridization. The carbonitrile side chain (C9–C12–N1) is almost linear, with the angle around central carbon atom being 179.6 (2)°. The geometric parameters of the title molecule agrees well with those reported for similar structures (Karthikeyan *et al.,* 2011, Loghmani-Khouzani *et al.,* 2009).

The crystal packing features C—H···O hydrogen bonds. Atom C3 in the molecule at *x, y, z*) directs its C—H bonds towards atom O4 at *1/2+x,1/2-y,1/2+z*, forming a C(8) chain along [101] (Fig. 2). Weak aromatic π—π interactions between the benzene rings of neighbouring molecules, with *Cg*—*Cg*^v^ distance of 3.887 (1) Å [Fig. 3; *Cg* is the centroid of the C1/C2/C4/C5/C6/C7 benzene ring, Symmetry code as in Fig. 3] are also observed.

### Experimental

To a solution of methyl 2-cyanoacetate (0.1 g, 1 mmol) in dichloromethane (5 ml), pyrrolidine (0.073 g, 1 mmol) was added and stirred well for 10 minutes. To this solution 6–nitrobenzo [d][1,3]dioxole–5–carbaldehyde (0.2 g, 1 mmol) was added and allowed to stir well for 12 h. After the completion of the reaction as evidenced by *TLC*, the reaction mixture was poured into 2 *N* HCl solution (10 ml) and extracted using 20 ml of dichloromethane. The organic layer thus obtained was concentrated under reduced pressure. Column purification (silica gel, mesh size: 60–120) of the crude mixture using 10% ethyl acetate in hexanes successfully provided the desired title compound in 92% yield (0.25 g).

### Refinement

All the H atoms were positioned geometrically with C–H = 0.93–0.97 Å and constrained to ride on their parent atom, with *U*_iso_(H) =1.5*U*_eq_ for methyl H atoms and 1.2*U*_eq_(C) for other H atoms.

### Crystal data

**Table:**

C_12_H_8_N_2_O_6_	*F*(000) = 568
*M**_r_* = 276.20	*D*_x_ = 1.503 Mg m^−^^3^
Monoclinic, *P*2_1_/*n*	Mo *K*α radiation, λ = 0.71073 Å
Hall symbol: -P 2yn	Cell parameters from 3589 reflections
*a* = 10.8191 (9) Å	θ = 2.3–30.1°
*b* = 7.3220 (6) Å	µ = 0.12 mm^−^^1^
*c* = 15.4133 (13) Å	*T* = 293 K
β = 91.691 (2)°	Block, colourless
*V* = 1220.47 (18) Å^3^	0.23 × 0.22 × 0.17 mm
*Z* = 4	

### Data collection

**Table:**

Bruker APEXII CCD diffractometer	3567 independent reflections
Radiation source: fine-focus sealed tube	2286 reflections with *I* > 2σ(*I*)
Graphite monochromator	*R*_int_ = 0.027
Detector resolution: 10.0 pixels mm^-1^	θ_max_ = 30.1°, θ_min_ = 2.3°
ω scans	*h* = −15→14
Absorption correction: multi-scan (*SADABS*; Sheldrick, 1996)	*k* = −6→10
*T*_min_ = 0.972, *T*_max_ = 0.979	*l* = −21→19
14190 measured reflections	

### Refinement

**Table:**

Refinement on *F*^2^	Primary atom site location: structure-invariant direct methods
Least-squares matrix: full	Secondary atom site location: difference Fourier map
*R*[*F*^2^ > 2σ(*F*^2^)] = 0.046	Hydrogen site location: inferred from neighbouring sites
*wR*(*F*^2^) = 0.129	H-atom parameters constrained
*S* = 1.03	*w* = 1/[σ^2^(*F*_o_^2^) + (0.0569*P*)^2^ + 0.1956*P*] where *P* = (*F*_o_^2^ + 2*F*_c_^2^)/3
3567 reflections	(Δ/σ)_max_ = 0.001
182 parameters	Δρ_max_ = 0.20 e Å^−^^3^
0 restraints	Δρ_min_ = −0.18 e Å^−^^3^

### Special details

**Table:**

Geometry. All esds (except the esd in the dihedral angle between two l.s. planes) are estimated using the full covariance matrix. The cell esds are taken into account individually in the estimation of esds in distances, angles and torsion angles; correlations between esds in cell parameters are only used when they are defined by crystal symmetry. An approximate (isotropic) treatment of cell esds is used for estimating esds involving l.s. planes.
Refinement. Refinement of F^2^ against ALL reflections. The weighted R-factor wR and goodness of fit S are based on F^2^, conventional R-factors R are based on F, with F set to zero for negative F^2^. The threshold expression of F^2^ > 2sigma(F^2^) is used only for calculating R-factors(gt) etc. and is not relevant to the choice of reflections for refinement. R-factors based on F^2^ are statistically about twice as large as those based on F, and R- factors based on ALL data will be even larger.

### Fractional atomic coordinates and isotropic or equivalent isotropic displacement parameters (Å^2^)

**Table:**

	*x*	*y*	*z*	*U*_iso_*/*U*_eq_	
C1	0.34825 (14)	0.0705 (2)	0.14286 (9)	0.0415 (3)	
H1	0.3194	0.0163	0.1929	0.050*	
C2	0.44834 (14)	0.1836 (2)	0.14611 (9)	0.0408 (3)	
C3	0.60882 (16)	0.3581 (3)	0.18676 (11)	0.0560 (4)	
H3A	0.5974	0.4774	0.2126	0.067*	
H3B	0.6912	0.3149	0.2026	0.067*	
C4	0.49334 (13)	0.26561 (19)	0.07304 (10)	0.0395 (3)	
C5	0.43953 (14)	0.2399 (2)	−0.00694 (9)	0.0406 (3)	
H5	0.4697	0.2955	−0.0563	0.049*	
C6	0.33678 (13)	0.12542 (19)	−0.01065 (8)	0.0372 (3)	
C7	0.28993 (13)	0.03817 (19)	0.06181 (9)	0.0375 (3)	
C8	0.18022 (14)	−0.0796 (2)	0.05853 (9)	0.0428 (3)	
H8	0.1115	−0.0384	0.0266	0.051*	
C9	0.17142 (13)	−0.2415 (2)	0.09786 (9)	0.0414 (3)	
C10	0.05270 (16)	−0.3443 (2)	0.09545 (10)	0.0481 (4)	
C11	−0.0446 (2)	−0.6111 (3)	0.14488 (14)	0.0865 (8)	
H11A	−0.0937	−0.5637	0.1906	0.130*	
H11B	−0.0215	−0.7349	0.1579	0.130*	
H11C	−0.0917	−0.6077	0.0911	0.130*	
C12	0.27569 (16)	−0.3259 (2)	0.14154 (11)	0.0484 (4)	
N1	0.35768 (15)	−0.3935 (2)	0.17628 (12)	0.0710 (5)	
N2	0.28260 (12)	0.09294 (19)	−0.09677 (8)	0.0472 (3)	
O1	0.51784 (12)	0.23313 (16)	0.21683 (7)	0.0566 (3)	
O2	0.59385 (11)	0.36932 (17)	0.09442 (7)	0.0549 (3)	
O3	0.21209 (14)	−0.03492 (18)	−0.10801 (8)	0.0666 (4)	
O4	0.31029 (14)	0.1961 (2)	−0.15503 (8)	0.0796 (5)	
O5	−0.03967 (12)	−0.2902 (2)	0.06023 (9)	0.0704 (4)	
O6	0.06541 (12)	−0.50122 (16)	0.13725 (8)	0.0589 (3)	

### Atomic displacement parameters (Å^2^)

**Table:**

	*U*^11^	*U*^22^	*U*^33^	*U*^12^	*U*^13^	*U*^23^
C1	0.0503 (9)	0.0418 (8)	0.0322 (6)	−0.0033 (7)	0.0004 (6)	0.0053 (6)
C2	0.0479 (8)	0.0384 (8)	0.0357 (7)	0.0000 (6)	−0.0082 (6)	0.0024 (6)
C3	0.0527 (10)	0.0612 (10)	0.0530 (9)	−0.0109 (8)	−0.0154 (8)	0.0049 (8)
C4	0.0381 (7)	0.0362 (7)	0.0440 (7)	−0.0009 (6)	−0.0040 (6)	0.0060 (6)
C5	0.0440 (8)	0.0405 (7)	0.0374 (7)	0.0013 (6)	0.0008 (6)	0.0083 (6)
C6	0.0411 (7)	0.0374 (7)	0.0327 (6)	0.0029 (6)	−0.0050 (6)	0.0023 (6)
C7	0.0390 (7)	0.0346 (7)	0.0389 (7)	0.0005 (6)	−0.0001 (6)	0.0027 (6)
C8	0.0398 (8)	0.0483 (8)	0.0403 (7)	−0.0002 (7)	−0.0014 (6)	−0.0014 (6)
C9	0.0381 (7)	0.0464 (8)	0.0400 (7)	−0.0044 (6)	0.0054 (6)	−0.0008 (6)
C10	0.0468 (9)	0.0586 (10)	0.0395 (7)	−0.0135 (8)	0.0091 (7)	−0.0080 (7)
C11	0.0961 (16)	0.0936 (16)	0.0707 (13)	−0.0623 (14)	0.0181 (11)	−0.0093 (12)
C12	0.0437 (9)	0.0436 (8)	0.0584 (9)	−0.0069 (7)	0.0090 (7)	0.0084 (7)
N1	0.0541 (9)	0.0636 (10)	0.0951 (13)	0.0040 (8)	0.0009 (9)	0.0224 (9)
N2	0.0523 (8)	0.0526 (8)	0.0364 (6)	−0.0010 (7)	−0.0047 (6)	0.0011 (6)
O1	0.0685 (8)	0.0590 (7)	0.0412 (6)	−0.0174 (6)	−0.0175 (5)	0.0084 (5)
O2	0.0505 (7)	0.0605 (7)	0.0529 (6)	−0.0165 (6)	−0.0125 (5)	0.0109 (6)
O3	0.0882 (9)	0.0585 (8)	0.0520 (7)	−0.0219 (7)	−0.0152 (6)	−0.0052 (6)
O4	0.0919 (10)	0.1096 (12)	0.0367 (6)	−0.0364 (9)	−0.0120 (6)	0.0199 (7)
O5	0.0437 (7)	0.0963 (10)	0.0708 (8)	−0.0157 (7)	−0.0048 (6)	0.0024 (7)
O6	0.0640 (8)	0.0560 (7)	0.0575 (7)	−0.0254 (6)	0.0143 (6)	−0.0048 (6)

### Geometric parameters (Å, º)

**Table:**

C1—C2	1.363 (2)	C7—C8	1.467 (2)
C1—C7	1.403 (2)	C8—C9	1.336 (2)
C1—H1	0.9300	C8—H8	0.9300
C2—O1	1.3553 (17)	C9—C12	1.436 (2)
C2—C4	1.378 (2)	C9—C10	1.488 (2)
C3—O2	1.430 (2)	C10—O5	1.191 (2)
C3—O1	1.431 (2)	C10—O6	1.323 (2)
C3—H3A	0.9700	C11—O6	1.444 (2)
C3—H3B	0.9700	C11—H11A	0.9600
C4—O2	1.3589 (18)	C11—H11B	0.9600
C4—C5	1.361 (2)	C11—H11C	0.9600
C5—C6	1.392 (2)	C12—N1	1.136 (2)
C5—H5	0.9300	N2—O3	1.2169 (17)
C6—C7	1.3949 (19)	N2—O4	1.2177 (17)
C6—N2	1.4547 (18)		
			
C2—C1—C7	118.11 (13)	C1—C7—C8	118.17 (13)
C2—C1—H1	120.9	C9—C8—C7	125.06 (14)
C7—C1—H1	120.9	C9—C8—H8	117.5
O1—C2—C1	127.85 (13)	C7—C8—H8	117.5
O1—C2—C4	109.86 (13)	C8—C9—C12	121.93 (14)
C1—C2—C4	122.29 (13)	C8—C9—C10	120.68 (14)
O2—C3—O1	107.47 (12)	C12—C9—C10	117.36 (14)
O2—C3—H3A	110.2	O5—C10—O6	125.79 (15)
O1—C3—H3A	110.2	O5—C10—C9	123.73 (16)
O2—C3—H3B	110.2	O6—C10—C9	110.48 (15)
O1—C3—H3B	110.2	O6—C11—H11A	109.5
H3A—C3—H3B	108.5	O6—C11—H11B	109.5
O2—C4—C5	127.95 (13)	H11A—C11—H11B	109.5
O2—C4—C2	110.18 (13)	O6—C11—H11C	109.5
C5—C4—C2	121.88 (13)	H11A—C11—H11C	109.5
C4—C5—C6	116.13 (13)	H11B—C11—H11C	109.5
C4—C5—H5	121.9	N1—C12—C9	179.57 (18)
C6—C5—H5	121.9	O3—N2—O4	122.63 (13)
C5—C6—C7	123.46 (13)	O3—N2—C6	119.32 (13)
C5—C6—N2	115.83 (12)	O4—N2—C6	118.05 (13)
C7—C6—N2	120.64 (13)	C2—O1—C3	106.34 (11)
C6—C7—C1	118.12 (13)	C4—O2—C3	105.99 (12)
C6—C7—C8	123.67 (13)	C10—O6—C11	116.71 (16)
			
C7—C1—C2—O1	179.97 (15)	C7—C8—C9—C12	−6.3 (2)
C7—C1—C2—C4	−0.1 (2)	C7—C8—C9—C10	175.68 (13)
O1—C2—C4—O2	0.51 (18)	C8—C9—C10—O5	−0.7 (2)
C1—C2—C4—O2	−179.41 (14)	C12—C9—C10—O5	−178.88 (16)
O1—C2—C4—C5	−179.43 (13)	C8—C9—C10—O6	179.10 (13)
C1—C2—C4—C5	0.7 (2)	C12—C9—C10—O6	0.96 (19)
O2—C4—C5—C6	179.86 (14)	C5—C6—N2—O3	163.45 (14)
C2—C4—C5—C6	−0.2 (2)	C7—C6—N2—O3	−13.6 (2)
C4—C5—C6—C7	−0.7 (2)	C5—C6—N2—O4	−16.5 (2)
C4—C5—C6—N2	−177.74 (13)	C7—C6—N2—O4	166.40 (15)
C5—C6—C7—C1	1.2 (2)	C1—C2—O1—C3	−178.04 (16)
N2—C6—C7—C1	178.11 (13)	C4—C2—O1—C3	2.05 (18)
C5—C6—C7—C8	178.73 (13)	O2—C3—O1—C2	−3.74 (18)
N2—C6—C7—C8	−4.4 (2)	C5—C4—O2—C3	177.10 (16)
C2—C1—C7—C6	−0.8 (2)	C2—C4—O2—C3	−2.83 (17)
C2—C1—C7—C8	−178.41 (13)	O1—C3—O2—C4	4.01 (18)
C6—C7—C8—C9	135.22 (16)	O5—C10—O6—C11	−3.8 (2)
C1—C7—C8—C9	−47.3 (2)	C9—C10—O6—C11	176.34 (14)

### Hydrogen-bond geometry (Å, º)

**Table:**

*D*—H···*A*	*D*—H	H···*A*	*D*···*A*	*D*—H···*A*
C3—H3*B*···O4^i^	0.97	2.51	3.247 (2)	132

Symmetry code: (i) *x*+1/2, −*y*+1/2, *z*+1/2.
